# Genomic and morphological characterization of *Knufia obscura* isolated from the Mars 2020 spacecraft assembly facility

**DOI:** 10.1038/s41598-024-61115-1

**Published:** 2024-05-28

**Authors:** Atul Munish Chander, Marcus de Melo Teixeira, Nitin K. Singh, Michael P. Williams, Ceth W. Parker, Patrick Leo, Jason E. Stajich, Tamas Torok, Scott Tighe, Christopher E. Mason, Kasthuri Venkateswaran

**Affiliations:** 1 Biotechnology and Planetary Protection Group, Jet Propulsion Laboratory, California Institute of Technology, M/S 89-2, 4800 Oak Grove Dr., Pasadena, CA 91109 USA; 2 Pathogen and Microbiome Institute, Northern Arizona University, Flagstaff, AZ USA; 3 School of Medicine, University of Brasilia, Brasília, DF Brazil; 4 Department of Microbiology and Plant Pathology, University of CA–Riverside, Riverside, CA USA; 5 Ecology Department, Lawrence Berkeley National Laboratory, Berkeley, CA USA; 6 Vermont Integrative Genomics Lab, University of Vermont, Burlington, VT USA; 7 WorldQuant Initiative for Quantitative Prediction, Weill Cornell Medicine, 1305 York Avenue, Room Y-13.15, New York, NY 10021 USA

**Keywords:** Black fungi, *Extremophile*, *Trichomeriaceae*, *Chaetothyriales*, Genomics, Fungal genomics, Biodiversity

## Abstract

Members of the family *Trichomeriaceae,* belonging to the *Chaetothyriales* order and the *Ascomycota* phylum, are known for their capability to inhabit hostile environments characterized by extreme temperatures, oligotrophic conditions, drought, or presence of toxic compounds. The genus *Knufia* encompasses many polyextremophilic species. In this report, the genomic and morphological features of the strain FJI-L2-BK-P2 presented, which was isolated from the Mars 2020 mission spacecraft assembly facility located at the Jet Propulsion Laboratory in Pasadena, California. The identification is based on sequence alignment for marker genes, multi-locus sequence analysis, and whole genome sequence phylogeny. The morphological features were studied using a diverse range of microscopic techniques (bright field, phase contrast, differential interference contrast and scanning electron microscopy). The phylogenetic marker genes of the strain FJI-L2-BK-P2 exhibited highest similarities with type strain of *Knufia obscura* (CBS 148926^T^) that was isolated from the gas tank of a car in Italy. To validate the species identity, whole genomes of both strains (FJI-L2-BK-P2 and CBS 148926^T^) were sequenced, annotated, and strain FJI-L2-BK-P2 was confirmed as *K. obscura.* The morphological analysis and description of the genomic characteristics of *K. obscura* FJI-L2-BK-P2 may contribute to refining the taxonomy of *Knufia* species. Key morphological features are reported in this *K. obscura* strain, resembling microsclerotia and chlamydospore-like propagules. These features known to be characteristic features in black fungi which could potentially facilitate their adaptation to harsh environments.

## Introduction

The ability of microorganisms to survive and proliferate in extreme environments is facilitated by numerous characteristics; chief among them is their capacity to withstand the competition and stress presented by their natural habitats^1,2^. These microorganisms are capable of adapting to both physical and chemical stressors via phenotypic plasticity^3,4^. Some fungi, for example, can survive under harsh conditions by developing specialized morphological and reproductive structures, such as cysts, conidia, or spores^5,6^. The detection of resilient black fungi persisting under oligotrophic conditions in cleanrooms of spacecraft assembly facilities (SAFs) may pose a risk of forward contamination to planets targeted for space exploration. If these fungi inadvertently hitch a ride on the components of planned space missions designed for life-detection efforts, they could potentially compromise the integrity of such explorations.

Certain fungi, categorized as polyextremotolerant, inhabit environments that are inhospitable to most other organisms, due to factors such as extreme temperatures, desiccation, radiation, chemical exposure, and nutrient scarcity^7^. Other polyextremophilic fungi have been isolated from a diverse range of environments, including areas with high concentrations of monoaromatic and volatile hydrocarbons, gasoline, and diesel fuel^8–10^. Moreover, certain species can thrive in the presence of polyphenols, shikimic acids, monoaromatic compounds, depsides, depsidones, diphenyl ethers, and dibenzofurans^11^.

The members of the genus *Knufia* are black fungi within the family *Trichomeriaceae*, order *Chaetothyriales,* and phylum *Ascomycota*^12^. *Chaetothyriales* also encompasses other families of black fungi, namely *Herpotrichiellaceae, Cyphellophoraceae,* and *Coccodiniaceae,* which are likewise regarded as polyextremotolerant^13,14^. Species within the family *Trichomeriaceae* rock-inhabiting fungi colonize rock surfaces^15,16^. They have also been detected on stone sculptures^17,18^. These fungi demonstrate physiological versatility that allows them to survive on human-made structures and in extreme habitats, such as cold and hot deserts, salterns, caves, dishwashers, roofs, solar panels, and tombs. The black fungi of the order *Chaetothyriales* are distinctively known for producing highly specialized polymers e.g., polyphenols (including melanins), chitin, and glucans. They also produce enzymes, such as cytochrome P450, glutathione S-transferases, catalases, and superoxide dismutases. Furthermore, they possess an efficient DNA repair system that shields them from a wide variety of toxic compounds and radiation^19–22^.

*Knufia* species are melanized fungi that grow slowly and display limited morphological differentiation. Given their highly convergent morphology, molecular analyses are often employed for their identification and phylogeny^23^. Recently, *Knufia* was established as a separate genus within the family, a decision supported by both molecular and morphological data^24,25^. Elucidating the genomic and phenotypic characteristics of a *Knufia* species enhances our understanding of the adaptive strategies they employ in extreme environments. It is important to note that as of now, only seven genomes of the family *Trichomeriaceae* are available in databases, such as the National Center for Biotechnology Information (NCBI), FungiDB, or Mycocosm, with the majority of them lacking genome annotation.

The objective of this study was to characterize the strain FJI-L2-BK-P2, which was isolated from the SAF used for the Mars 2020 mission at the National Aeronautics and Space Administration's (NASA) Jet Propulsion Laboratory (JPL). The morphological features of strain FJI-L2-BK-P2 were compared to those of the type strain of *Knufia obscura* CBS 148926^T^ that was isolated from a the gasoline tank of a car in Italy^26^. The whole genome sequences (WGS) for both strains of *K. obscura* were generated, assembled, annotated, and subsequently used for multi-locus sequence analysis (MLSA) and WGS phylogeny. Additionally, we performed a thorough analysis of those morphological features in *K. obscura* FJI-L2-BK-P2, which might be potentially significant for the survival and adaptation of *Knufia* species in harsh environments. The genomic and morphological characteristics of the *K. obscura* strains discussed in this study further refine both molecular and traditional taxonomical identification of other *Knufia* species.

The results of this study showed that FJI-L2-BK-P2, characterized by septate hyphae, unique conidiophores, and chlamydospores, genetically aligns closely with *K. obscura* CBS 148926^T^ based on molecular phylogeny analyses. In addition, FJI-L2-BK-P2 withstands extreme UV-C, indicating its importance for NASA planetary protection and provides insight into the resilience of this black fungus.

## Results

In previous work^27^, we isolated 65 strains from two NASA SAFs and by sequencing the internal transcribed spacer (ITS) region 28 unique fungal species were identified, including a strain belonging to the genus *Knufia*. The ITS sequence of this *Knufia* isolate, FJI-L2-BK-P2, did not align with any known fungal species until 2022 when *K. obscura* was described by molecular phylogeny and published as a new species^26^. Subsequent molecular analyses revealed that the strain FJI-L2-BK-P2 was found to be closely related to the type strain of *K. obscura*^26^*.*

### Morphology

**Figure 1:**
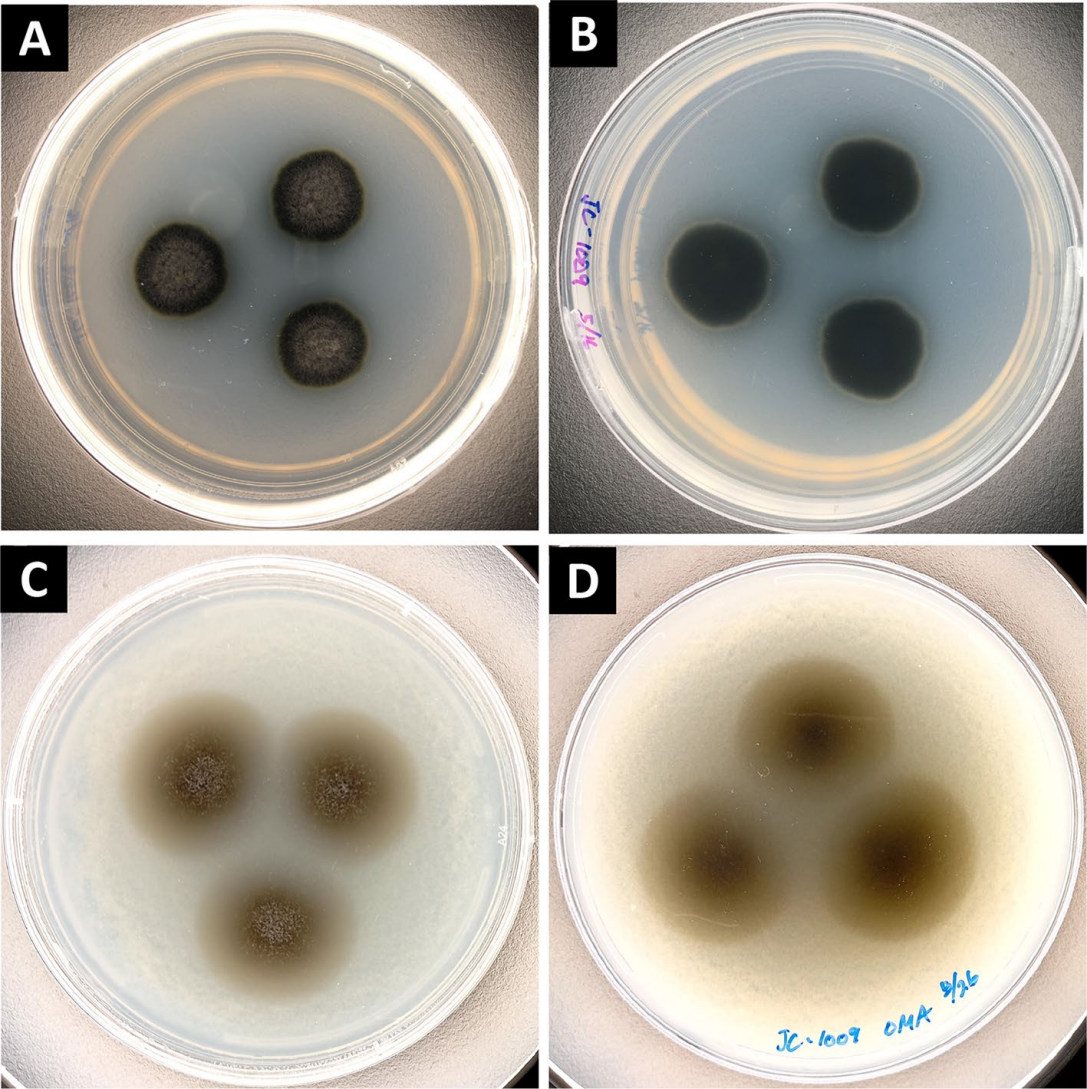
Colony morphology of *Knufia obscura* FJI-L2-BK-P2. Colonies after incubation at 25 °C on PDA medium (14 days after inoculation; **A**,**B**) and on OMA medium (21 days after inoculation; **C**,**D**). (**A**) and (**C**) are front side of the plates whereas (**B**) and (**D**) are back side of the plates.

Morphological features of *K. obscura* FJI-L2-BK-P2 are shown in Fig. 1. On day 14, colonies on potato dextrose agar (PDA) were irregular, ~ 20 mm in diameter, dark black with yellow pigmentation near the periphery. The center of colony appeared yellowish brown and cottony (Fig. 1A). The reverse side of colonies on PDA appeared dark black with yellow pigmentation that encircled the entire colony (Fig. 1B). On day 21, colonies on oatmeal agar (OMA) were more regular (circular) in shape, ~ 25 mm in diameter, dark brown with cottony appearance in the center, and light brown on the periphery (Fig. 1C). The back of colonies on OMA appeared dark brown in the center and light brown on the periphery (Fig. 1D).

**Figure 2:**
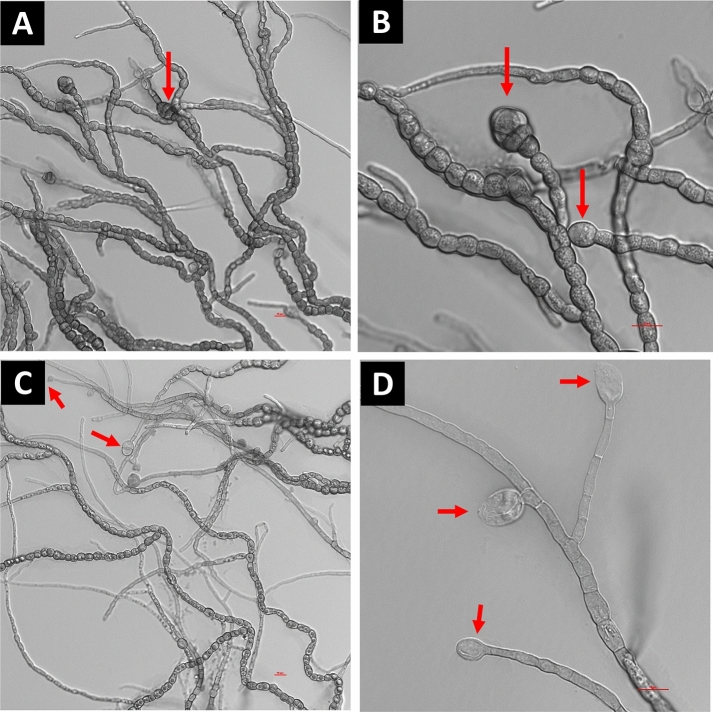
Microscopic features shown by DIC microscopy for *Knufia obscura* FJI-L2-BK-P2. (**A**,**B**). Mature hyphae with multicellular globose apex containing large pear shaped chlamydospores. (**C**,**D**) Conidia and conidiophores (arrows). Image (**A**) and (**C**) are at ×400 total magnification and image (**B**) and (**D**) are taken at ×1000 total magnification.

**Figure 3:**
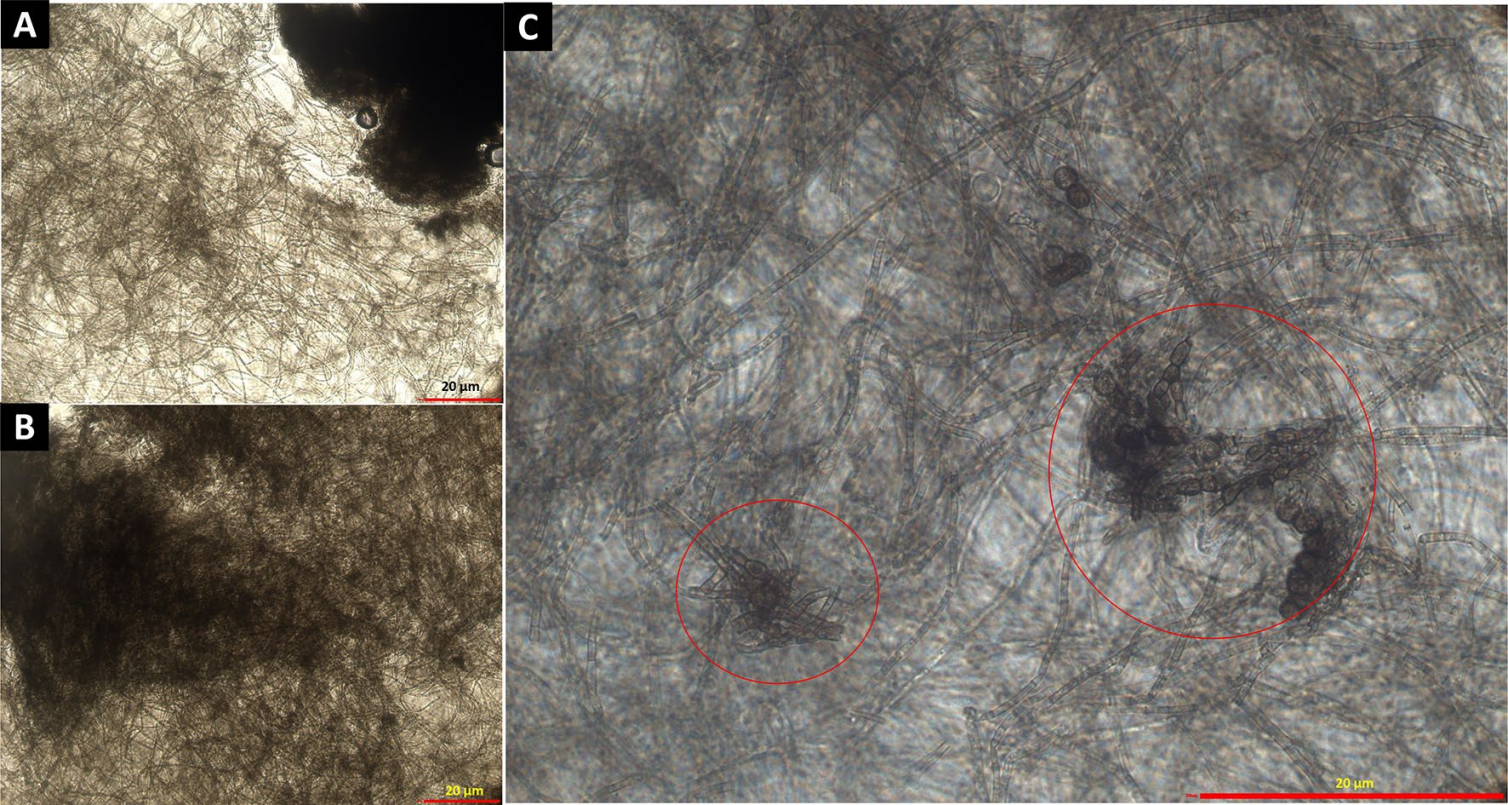
Bright field (**A**,**B**) and phase contrast microscopy (**C**) provides evidences for presence of microsclerotia in *Knufia obscura* FJI-L2-BK-P2: (**A**,**B**) Extensive hyphal branching and hyphae intermingle to form dense microsclerotia type network. (**C**) Closely attached multi-celled chlamydospores intermingled form microsclerotia indicated by using phase contrast microscopy. Images (**A**) and (**B**) are at ×200 and image (**C**) is at ×1000 total magnification.

**Figure 4:**
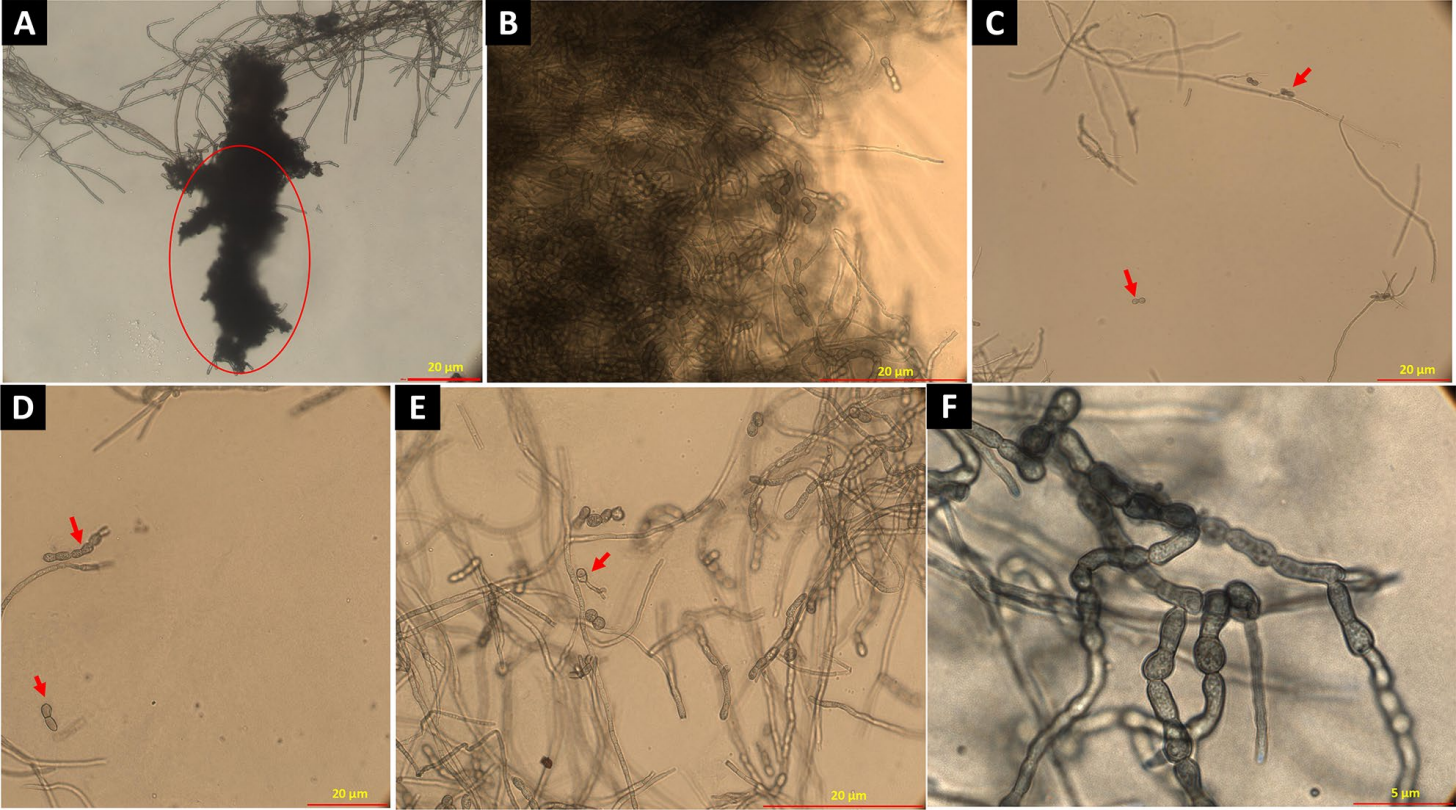
Bright field microscopy revealed chlamydospore featured mature microsclerotia. (**A**) Microsclerotium forms a dense clump of biomass (encircled). (**B**) Microsclerotia containing intermingled arthroconidia and chlamydospores. (**C**,**D**) Arthroconidia released (arrow). (**E**) Germinating conidia (arrow). (**F**,**G**) Arthroconidia. Images (**A**) and (**C**) are taken at ×200, (**B**), (**D**) and (**E**) are taken at ×400 and image (**F**) is taken at ×1000 total magnification.

**Figure 5:**
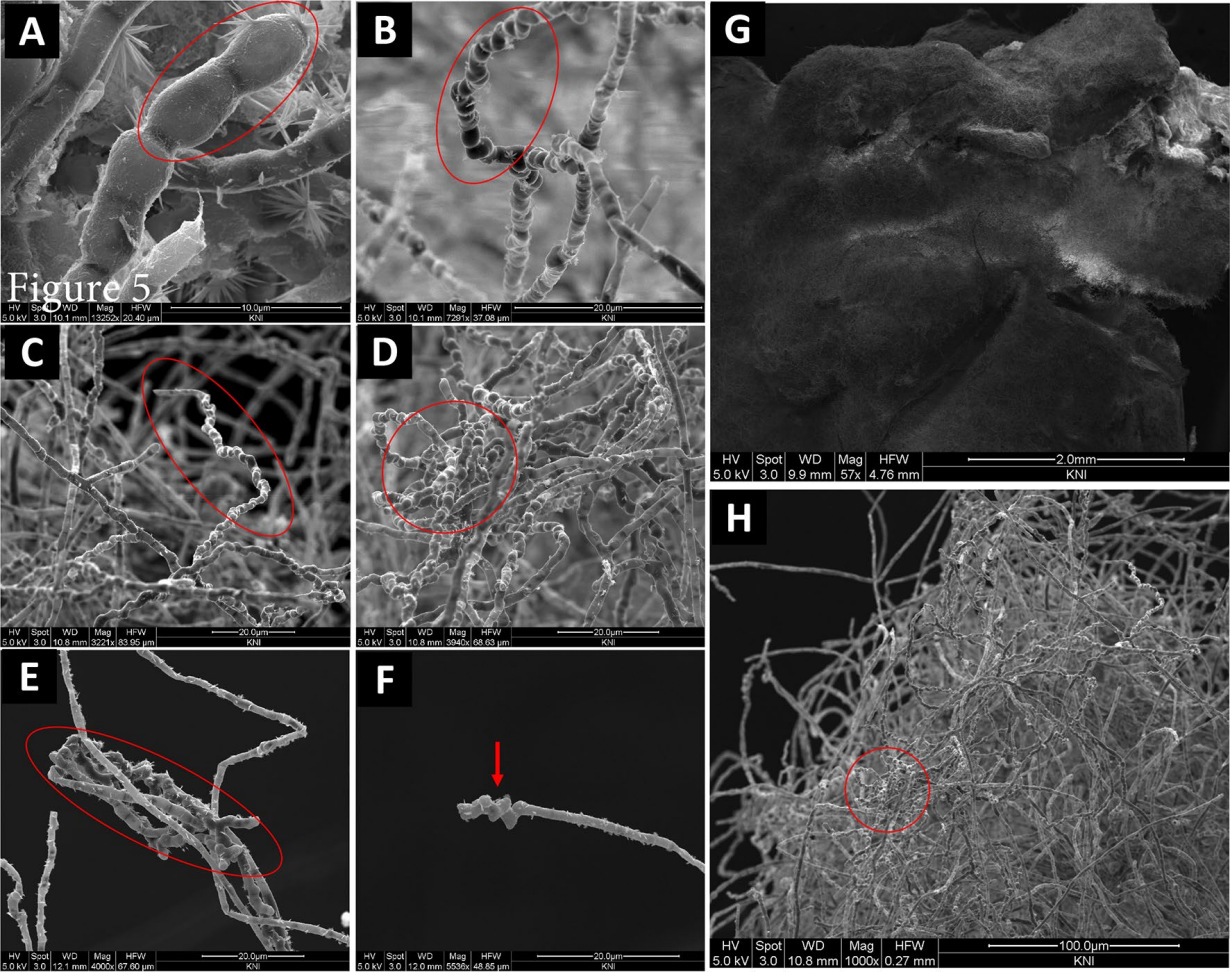
SEM images. (**A**–**C**) Arthroconidia (encircled). (**D**,**E**) Microsclerotia. (**F**) Chlamydospore/chlamydospore like propagules. (**G**) Microsclerotia network creates dense surface layer over the fungal colony. (**H**) Microsclerotia.

Differential interference contrast (DIC) microscopic images of *K. obscura* FJI-L2-BK-P2 are shown in a number of figures. Hyphae and conidiophores are septate. Mature hyphae exhibit swollen structure and often appear like arthroconidia prior to separation, as recently seen in genus *Pasadenomyces* (Fig. 2A,B, swollen forms, Figs. 3 and 4F,G)^28^. Conidiophores are smooth walled (Fig. 2C) unlike the regular hyphae and seem to originate from mature hyphae (Fig. 2A). FJI-L2-BK-P2 has distinguishing features from other *Knufia* species by having swollen chlamydospore propagules intercalary and pear-shaped, ~ 30 µm in length, at apex of the mature hyphae (Figs. 2A,B and 5F). Conidia (oval; ~ 20 µm in diameter) are noticed on the conidiophores (Fig. 2C,D). Conidiogenous cells are monoblastic. Conidiophores are light colored unlike the dark colored chlamydogenous hyphae. Conidiophores are directly differentiated to form conidiogenous cells, which produce single conidia (Fig. 2D). In contrast, conidiophores also differentiate into branches of variable length and apical cells give rise to bulbous conidia. In short, conidia are either attached directly onto the conidiophore or on the new branch (Fig. 2D). So far, such conidiophores and conidia formation have not been reported in strains of *Knufia* species (or these features remained undetected or not reported). In addition, the pear-shaped chlamydospore observed at terminal ends and intercalary positions is a distinguishing morphological feature of FJI-L2-BK-P2 compared to the type strain CBS 148926^T^. These structures are also analogous to the microsclerotia reported in members of *Phialocephala*^29^. Two types of microsclerotia formation were observed in this strain (Figs. 3 and 4). Vegetative hyphae containing arthroconidia and chlamydogenous hyphae intermingle separately or together with each other to form dense/hard microsclerotia (Fig. 5).

### UV-C Irradiation Resistance of *Knufia obscura* FJI-L2-BK-P2

Survival of the fungus was assessed as a binary outcome (survival: yes/no) across a range of UV-C exposure doses. Growth was observed for K. obscura FJI-L2-BK-P2 after exposure to UV-C doses ranging from 1,000-9,999 J/m2, confirming the strain’s resistant to UV-C (up to doses of 9,999 J/m2). This assay was based on a qualitative response across all treatments.

### Variations of several molecular markers among closely related *Knufia* species

Sequences of two marker genes (ITS and large subunit [LSU] of rRNA) of FJI-L2-BK-P2 with the type strain of *K. obscura* were compared*.* The LSU sequence comparison showed that strain FJI-L2-BK-P2 has maximum sequence identity of 99.64% with *K. obscura* CBS 148926^T^, followed by *K. hypolithi* (98.48%). This data suggests that strain FJI-L2-BK-P2 is genetically related to *K. obscura* CBS 148926^T^. Similarly, ITS sequence comparison shows that strain FJI-L2-BK-P2 has maximum sequence identity (99.46%) with *K. obscura* CBS 148926^T^*,* followed by *K. hypolithi* (98.48%). In the NCBI database, only the ITS and LSU sequences were available for the *K. obscura* CBS 148926^T^ type strain. To verify whether FJI-L2-BK-P2 is similar to or different from *K. obscura* CBS 148926^T^, we extracted other marker gene sequences from the WGS data for comparison. The sequence identities for *tef*1 (99.68%), actin (100%), *rpb*1 (100%), and ß-tubulin (100%) were notably high between the CBS 148926^T^ and NASA SAF strain and confirming that FJI-L2-BK-P2 strain is *K. obscura*.

### MLSA and whole genome phylogeny

**Figure 6:**
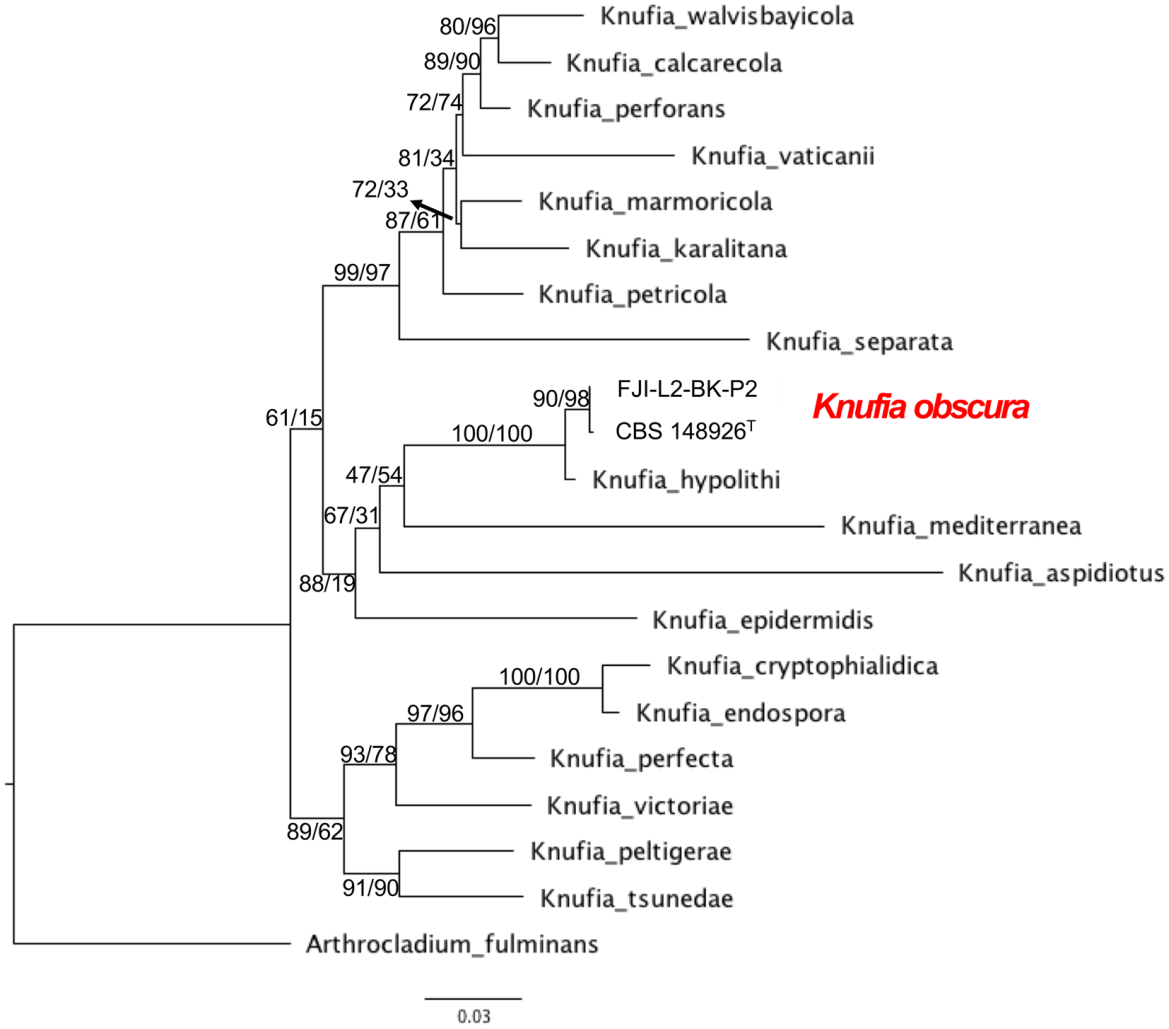
The MLSA of *Knufia obscura*. The genes ITS, LSU, *tef1, rpb1* and ß-tubulin were used to investigate phylogenetic placement of the *K. obscura* via ML tree on the IQTREE2 software.

**Figure 7:**
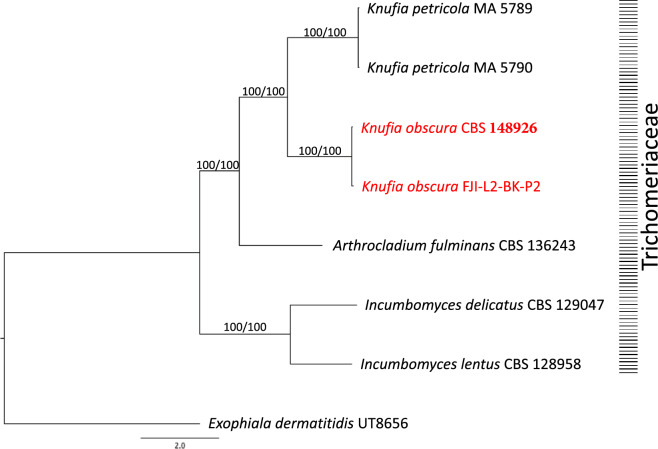
The WGS based phylogenomic analysis for *K. obscura.* Phylogenomic tree was constructed for *Knufia* strains whose genomes were available. Maximum-likelihood tree was constructed using the RAxML and ASTRAL software packages. *Exophiala dermatitidis* was set as the outgroup and the branches are proportional to the number of mutations. Branch fidelity used posterior probabilities, which were added next to the corresponding branches.

Next, we validated the results obtained by BLASTN using MLSA analysis of rDNA and nuclear ITS, LSU, *tef*1*, rpb*1, and ß-tubulin markers that are commonly used to diagnose species within *Chaetothyriales*. As per MLSA, *K. hypolithi, K. mediterranea,* and *K. aspidiotus* were the closest species to *K. obscura* including the strain FJI-L2-BK-P2. According to our reconciled phylogenetic tree (or species tree), the *Knufia* clade is monophyletic and harbors at least 19 species (Fig. 6). The strain FJI-L2-BK-P2 is phylogenetically related to *K. obscura* CBS 148926^T^, thus, we diagnosed FJI-L2-BK-P2 as *K. obscura*. It is worth noting that *K. obscura* shares a common ancestor with *K. hypolithi* and forms a triad along with *K. mediterranea*. In addition to MLSA, the evolutionary relationships between *K. obscura* CBS 148926^T^ and FJI-L2-BK-P2 was characterized via WGS-based phylogeny (Fig. 7). We noticed that the strains are almost identical and map next to the *K. petricola* clade. The *Knufia* clade based on WGS is monophyletic and in agreement with MLSA phylogeny. The two *K. obscura* strains are partitioned with strong bootstrap/aLRT support. However, the two *K. obscura* isolates and *K. petricola* are similar in terms of branch length compared to the branch that separates the two species. The branches are proportional to the number of mutations and 1000 ultrafast bootstraps. Shimodaira–Hasegawa [SH]-like approximate likelihood ratio test (SH-aLRT) confirmed the branch support and values were added to each corresponding branch of the tree. The MLSA tree was rooted with *Arthrocladium fulminans* CBS 136243 and we included *Exophiala dermatitidis* UT8656 for the WGS phylogenetic tree. At the time of analyzing data for this paper, only 4 *Knufia* WGSs were available in public databases. Additional genomes are needed to better comprehend the evolutionary relationships among members of the *Knufia* genus, as well as within the *Trichomeriaceae* family.

### Genomic features of *Knufia obscura*

Assembled genomes of *K. obscura* FJI-L2-BK-P2 and CBS 148926^T^ were assessed and found to be ~ 91% complete with similar characteristics. A summary of genomic features and annotations of the two draft genomes is presented in Table 1. The genomes of FJI-L2-BK-P2 and CBS 148926^T^ were assembled into 140 and 54 contigs, respectively, and yielded 30.5 Mb and 30.3 Mb in size (Table 1). *K. obscura* appears to have 10 chromosomes according to the number of eukaryotic repeats found in the terminal end of the contigs (Table 1). The total number of genes, introns, and exons predicted (including mRNA and tRNA), the average of gene, protein, and exon lengths, as well as the annotation features, such as gene ontology (GO) terms, Interpro domains, EggNOG, MEROPS, CAZYmes, BUSCO, and secreted proteins were similar (Table 1).

**Table 1:** Genomic features of draft genome sequences.

Strains	*K. obscura* CBS 148,926	*K. obscura* FJI-L2-BK-P2
Sequencing method	Oxford Nanopore	Illumina
Contig counts	54	140
Total length	30,507,620	30,336,935
Min	4628	1007
Max	4,888,905	2,043,846
L50	6	12
N50	1,876,131	791,130
L90	19	45
N90	458,232	197,538
GC (%)	50.64	50.69
Genome completeness (%)	90.7	91.1
T2T scaffolds	0	1
Telomere Fwd	2	10
Telomere Rev	8	10
Genes	11,327	11,298
mRNA	11,248	11,220
tRNA	79	78
Average gene length	1519.2	1518.32
Total introns	9877	9858
Total exons	21,125	21,078
Average exon length	5519	532.84
Average protein length	482.91	482.8
GO terms	2697	2605
Interproscan	3735	3683
EggNOG	9301	9259
MEROPS	317	318
CAZYmes	325	322
BUSCO	3233	3263
Secretion	669	678

### Extraction of gene information from GenBank files

Apart from the above insights the number of gene identified in both the genomes were 2640 (Table S1) after eliminating all hypothetical proteins from the dataset.

#### Genomic clustering and shared features

**Figure 8:**
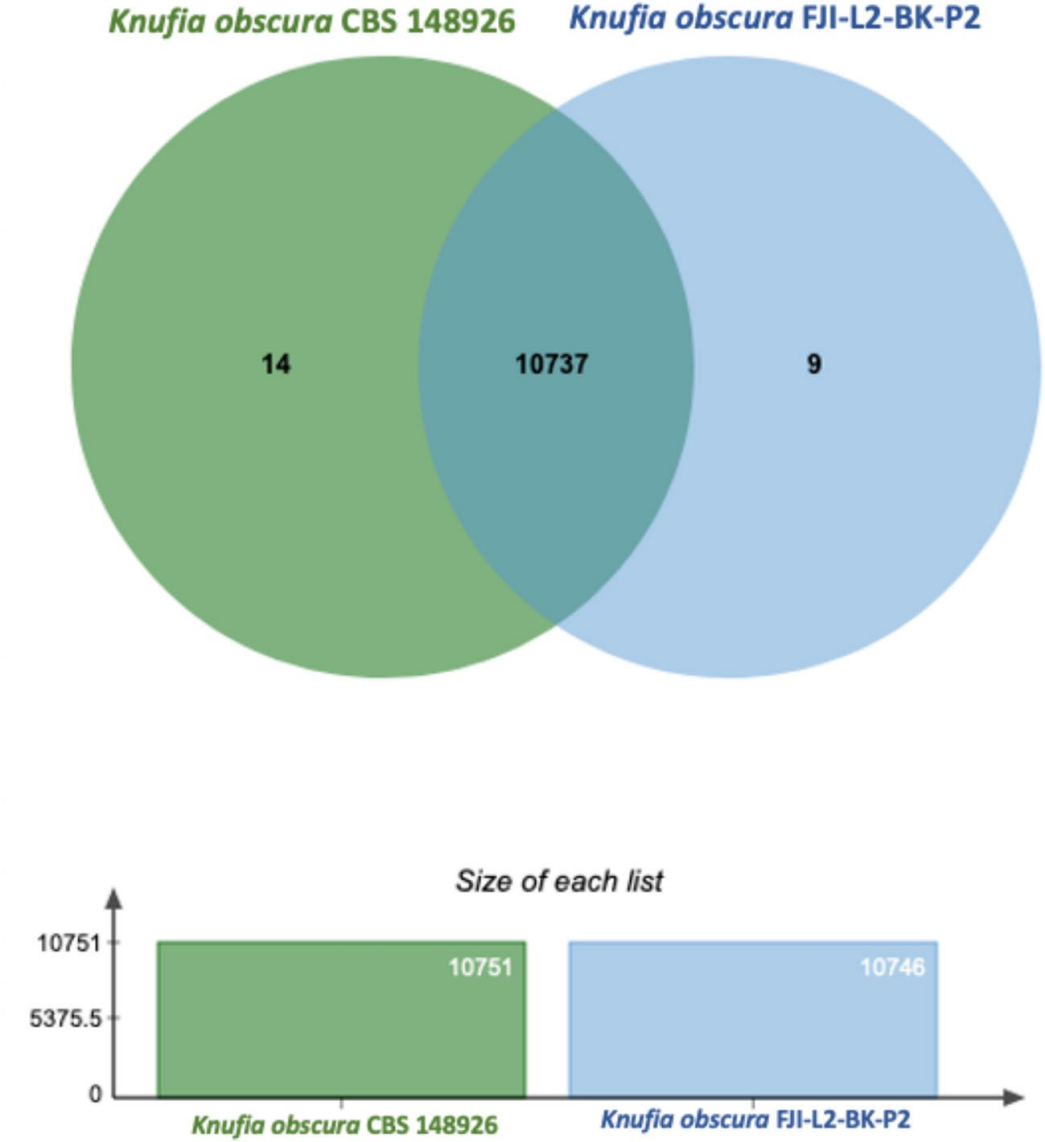
Shared and Unique Genomic Clusters between *Knufia obscura* strains*.* Venn diagram depicting the shared and unique genomic clusters between *Knufia obscura* FJI-L2-BK-P2 and CBS 148926^T^ strains. The figure presents a Venn diagram that illustrates the comparative genomic analysis of *Knufia obscura* strains, highlighting the shared and unique clusters of each.

Genomic clustering analysis demonstrated a substantial overlap in features between strains FJI-L2-BK-P2 and CBS 148926^T^. A shared 10,737 number of genes were identified in both the genomes, with 9 unique genes in FJI-L2-BK-P2 and 14 in strain CBS 148926^T^ (Fig. 8).

#### Functional annotation and gene ontology analysis

The GO analysis highlighted predicted differences in functional annotations between the strains (Table S1). Strain FJI-L2-BK-P2 has lost functions associated with RNA 3'-end processing, transferase activity, fatty acid beta-oxidation, and sphingolipid metabolism. In contrast, strain CBS 148926^T^ has gained functions in the urea catabolic process, metal ion transport, response to copper ion, ribosome biogenesis, and DNA replication processes (Table S2).

#### Gene ontology enrichment analysis

For strain CBS 148926^T^, gene ontology (GO) enrichment analysis revealed significant functional enrichments in various biological processes and molecular functions. Notably, CBS 148926^T^ showed enrichment in ubiquitin-dependent endocytosis (GO:0070886), urea metabolic process (GO:0019627), protein serine/threonine kinase activity (GO:0004674), and the Wnt signaling pathway (GO:0016055). In contrast, no significant GO enrichments were observed for strain FJI-L2-BK-P2.

### KEGG pathway annotations highlighting adaptability and survival

The KEGG pathway analysis for the two *K. obscura* strains reveals an extensive repertoire of metabolic and regulatory pathways, which are instrumental for adaptability and survival in diverse environments (Table S3).

#### Metabolic flexibility and energy conservation

Central to survival is metabolic flexibility, which in CBS 148926^T^ and FJI-L2-BK-P2, is evidenced by the annotation of carbohydrate metabolic processes (GO:0005975) and organic acid metabolic processes (GO:0006082; Table S2). These pathways suggest that both strains can utilize a range of substrates for energy and biomolecule synthesis. The presence of genes involved in the metabolism of alcohols and cellular aldehydes indicates a capacity for utilizing various organic compounds, which is particularly beneficial in nutrient-scarce environments^30^.

#### Stress response and detoxification

The xenobiotic metabolic process (GO:0006805) annotations indicate that both strains have developed mechanisms to cope with potentially harmful substances, which could include natural toxins or anthropogenic pollutants (Table S3). This capability is crucial for survival in extreme conditions, where exposure to damaging agents can be frequent^31^.

#### Genetic information processing

Nucleobase-containing compound metabolic processes (GO:0006139) and DNA replication (GO:0006260) are foundational for the growth and reproduction of these organisms (Table S2). The ability to replicate and repair DNA is essential for maintaining genomic integrity, especially in environments that can cause DNA damage, such as those with high radiation or desiccation^32^.

#### Cellular processes and organization

Annotations related to cellular component organization (GO:0016043) and biogenesis, such as ribosome biogenesis (GO:0042254), indicate that these strains invest in the maintenance and efficient assembly of cellular machinery (Table S3). This investment is crucial for rapid response and adaptation to environmental changes.

#### Transport mechanisms

The extensive annotations related to transport (GO:0006810), including ion transport (GO:0006811) and amino acid transport (GO:0006865) (Table S3), point to sophisticated mechanisms for the uptake of nutrients and disposal of waste products. Efficient transport systems are vital for survival in environments where nutrients may be patchy or in limited supply^33^.

#### Signal transduction and inter-organismal interactions

Signal transducer activity (GO:0004871) annotations highlight the ability of these strains to perceive and respond to their environment. Furthermore, the presence of genes associated with interspecies interactions (GO:0044419) suggests these strains may engage in complex ecological relationships, potentially including symbiosis or competition, which could be key to their survival in diverse microbial communities. 

## Discussion

Black fungi, known for their resilience, are found in some of the planet's most extreme environments, including the desolate cold of Antarctica, arid deserts, and the crushing depths of deep-sea hydrothermal vents^34^. These fungi have evolved a suite of adaptive mechanisms that enable them to cope with severe environmental stressors. Among these adaptations are pigmentation and specialized dormant structures like conidia and chlamydospores, which allow the fungi to withstand prolonged periods of desiccation^35^. In addition to these morphological adaptations, black fungi have developed unique metabolic pathways that support their survival in habitats where most life forms would struggle^21,36^.

*K. obscura*, a species within the resilient *Trichomeriaceae* family, is particularly adept at enduring extreme conditions, ranging from intense temperatures, drought, and nutrient scarcity to the presence of toxic compounds. Strains of *K. obscura* have been isolated from a variety of environments, which include human-made habitats (e.g., gasoline tank of the car)^26^, and natural environments (e.g., soils of Antarctica)^26^. The broad ecological range and adaptability suggest that *K. obscura* has the potential to be inadvertently present in sensitive environments, such as an SAF. The extremophilic capacity of *K. obscura* raises the question about its introduction, whether through environmental exposure or human-mediated transfer, and its survivability in the oligotrophic conditions of cleanrooms. To fully understand the risks posed by such extremophiles and their adaptability, further research into the genomic diversity of *Knufia* species is vital. Such investigations will provide insights into their potential to hitchhike spacecraft surfaces and endure the rigors of space.

To date, MycoBank lists 22 recognized species within the genus *Knufia*. Fungorum catalogues 21 species, omitting *K. aloicola* as reported in MycoBank. Genomic data for these species are scarce, with only the genomes of two *K. petricola* strains are currently available. The lack of WGS for the remaining 21 validly described *Knufia* species underscores the pressing need for extensive genetic research to clarify the evolutionary relationships within this genus and the wider *Trichomeriaceae* family.

In this study, we have not only described the morphological characteristics of the *K. obscura* strain FJI-L2-BK-P2 but also generated WGS for this strain along with the type strain CBS 148926^T^. Through rigorous phylogenetic analysis, employing MLSA and WGS methodologies, we have verified the phylogenetic affiliation of FJI-L2-BK-P2. The use of both Illumina and Oxford Nanopore Technology (ONT) sequencing methods was not to compare the DNA sequence composition accuracy between these methods, as their consistency is well-established^37^. The short-read sequencing (Illumina) of the ITS region of FJI-L2-BK-P2 showed a 99.46% identity to the corresponding region in the type strain CBS 148926^T^ that was sequenced using long read sequencing by the ONT. The MLSA and WGS-based analyses showed that both strains were phylogenetically identical.

The functional analysis predict substantially shared genomic features reflects the core genome conservation, while the presence of unique genes may be representative of strain-specific adaptations. The significant enrichment of ubiquitin-dependent endocytosis in strain CBS 148926^T^ predicts the advanced intracellular trafficking capability meant for contributing regulation of its cellular processes^38–40^. The identification of the predicted urea metabolic process can equip the species for an efficient nitrogen utilization pathway in nutrient-limited environments^41,42^. The presence of predicted protein serine/threonine kinase activity enrichment is indicative of it’s abilities to facilitate several cellular activities for growth and response to environmental stimuli^43^. The lack of significant GO enrichment and specific gene functions in FJI-L2-BK-P2 could also be due to the evolutionary trajectory of FJI-L2-BK-P2, or the sensitivity of the sequencing performed for it.

The KEGG pathway annotations underscore the genetic potential of strain CBS 148926^T^ and FJI-L2-BK-P2 for metabolic versatility, environmental resilience, and interactive complexity. The core genome's conservation highlights essential survival traits, while genomic differences underscore the species' adaptive flexibility and their potential utility in biotechnological applications. Further research into the ecological implications of the unique genomic traits may provide deeper understanding of the evolutionary strategies of these fungi.

A systematic review of available literature for *Knufia* species suggests that the morphological features of conidiophores and conidia observed in FJI-L2-BK-P2 have not been noticed or reported in other members of *Knufia,* whereas similar conidiophores and conidia formations were prominently described in several members of *Phialocephala,* a genus of the order *Helotiale*s^29^. Chlamydospore-like propagules were noticed in *K. hypolithi* in terminal and intercalary positions. However, these propagules found in *K. hypolithi* were subcylindrical to ovoid-ellipsoid^44^. These structures in *K. walvisbayicola* had subcylindrical to elongated ellipsoid-ovoid shape^44^. Arthroconidia were reported in *K. vaticanii* CBS 139722 and *K. marmoricola* CBS 139726. Enlarged, thick walled and brown bodies, containing endoconidia were reported in *K. marmoricola* CBS 139726 and *K. mediterranea* CBS 139721, which seem similar to chlamydospore-like propagules of strain FJI-L2-BK-P2^15^. Thick-walled rugose cells and terminal and intercalary multicellular bodies containing endoconidia were also observed in *K. karalitana* CBS 139720^45^, which seem similar to chlamydospore-like propagules described above by various authors. In *K. separata* CGMCC 3.17337, swollen cells like arthroconidia were reported globose to sub-globose. Multicellular bodies globose, ellipsoidal, or irregular, dark brown were also reported in *K. separata*, which seem similar to the endoconidia containing cells or chlamydospore-like propagules^45^. Microsclerotium-like structures, round to ovoid multicellular bodies, and chlamydospore-like propagules were also observed in *K. obscura* type strain CBS 148926^T^^26^. Despite minimal phylogenetic differences between the strains FJI-L2-BK-P2 and *K. obscura* CBS 148926^T^, the former displays a more varied morphology not found in the latter^26^. In conclusion, there are swollen cells in FJI-L2-BK-P2 (as seen in Fig. 4A–C), which bear similarity to those reported in *Knufia* species and are often referred to as arthroconidia^46^. More enlarged thick bodies containing endoconidia were also documented^15^ which are very similar to the chlamydospores seen in Fig. 2A,B (and might contain endoconidia inside). In phase contrast images we observed structures that were similar to chlamydospores and endoconidial forms (Fig. 3C; encircled). The terminal chlamydospore propagules in FJI-L2-BK-P2 were swollen, pear-shaped, and multicellular. Table 2 contains comparative information about all morphological features of *Knufia* strains discussed above.

**Table 2:** Mophological characteristics of *Knufia* strains discussed in this manuscript.

Strain	Colony appearance	Hyphal characteristics	Conidia/propagule features	Distinctive Traits	Optimal Growth Temperature
*K. obscura* FJI-L2-BK-P2	Irregular, dark black with yellow pigmentation, cottony center	Septate, mature hyphae swollen, resembling arthroconidia	Swollen chlamydospore propagules, oval conidia ~ 20 µm diameter	Resilient to UV-C radiation, extremophile characteristics	Not evaluated
*K. obscura* CBS 148,926	Slow growth, black with greenish-grey velvety mycelium	Smooth, branched, frequently anastomosed, subhyaline to pale brown	Multicellular bodies roundish to obovoid, brown to dark brown, thick-walled	Distinctly dark colony color, loosely reticulate network hyphae	25 °C
*K. victoriae*	Cauliflower-like, slow growth, black, few aerial mycelium	Mostly unbranched, toruloid, thin-walled	Roundish to subcylindrical multicellular bodies, muriform, brown to dark brown	Unique growth at low temperatures, characteristic multicellular bodies	10–15 °C
*K. karalitana*	Black, raised, deeply immersed into agar, short aerial hyphae	Moniliform, occasionally toruloid, brown	Spherical or bicellular and ellipsoidal conidia	Unique conidial formation, isolated from marble lion in Cagliari	Not Known
*K. marmoricola*	Black, raised, flat, glossy, velvety, grayish-brown aerial hyphae	Moniliform, branched, cylindrical, smooth, pale brown	Spherical, thick-walled, smooth conidia	Conidia formed by differentiation of terminal and intercalar hyphal cells	Not Known
*K. mediterranea*	Black, cerebriform, glossy, some greenish gray aerial mycelium	Scant filamentous, mostly moniliform, subhyaline to pale brown	Cells later become darker, roundish, mostly cylindrical, 1-septate	Distinct cerebriform colony appearance, moniliform hyphae	Not Known
*K. vaticanii*	Brownish-black, cerebriform, dense velvety aerial mycelium	Pale brown, finely guttulate, filamentous, branched	Arthroconidia terminal and intercalar, dark brown, thick-walled	Produces swollen arthroconidia, isolated from Vatican City State	Not Known

In some fungi, melanized multicellular globose structures similar to the chlamydospore-like propagules are part of firm, frequently rounded mass of hyphae. They are abundant in aerial mycelia and later form crust on colony surfaces with or without the addition of host tissue or soil^34^. They are termed microsclerotia, which survive longer periods in adverse habitats (up to 15 years)^29,47,48^. Formation of microsclerotia was observed in members of *Phialocephala*^29^, which appears to be a convergent morphological characteristic of adaptation. These structures were described differently by various taxonomists and a uniform protocol would be desirable to avoid confusion. It is noteworthy that microsclerotium-like structures were also observed in some recently defined novel fungal species from SAF^28^.

The environments from which *Knufia* species have been reported are extremely harsh (e.g., petroleum hydrocarbons in a gas tank), characterized by low nutrient availability (e.g., cleanrooms), high salinity, and severe temperature fluctuations—conditions that present significant challenges for most organisms^49^. These fungi have developed a number of mechanisms to cope with such conditions^50^. For instance, their conidia and other specialized structures, such as chlamydospores, allow these fungi to enter a dormant state for survival in desiccated conditions over long periods^51–53^. Moreover, the presence of chlamydospore-like propagules and microsclerotia observed in *K. obscura* FJI-L2-BK-P2, and in some members of *Phialocephala* have been reported to be adaptive traits that aid in tolerating adverse environments^34^.

The resilience of *K. obscura* to UV-C radiation is in line with the capabilities of extremophiles, which are adept at surviving in conditions considered extreme by human standards. The biochemical and physiological changes that extremophiles undergo, such as producing extremolytes and extremozymes, are essential for understanding the boundaries of life on Earth and are of significant interest in astrobiology. The survival mechanisms inferred from the genomes of *K. obscura* and its isolation highlight the necessity for rigorous decontamination procedures in SAFs to avoid potential biological contamination during space missions. Furthermore, understanding their survival mechanisms could potentially provide insights into how lifeforms persist in harsh and seemingly inhospitable conditions in extraterrestrial environments. Therefore, further research in black fungi and their survival strategies is crucial for the effective implementation of planetary protection measures.

## Materials and methods

### Sample collection and isolation of fungi

The strain FJI-L2-BK-P2 was isolated from samples collected at JPL-SAF in April 2018 as previously described^28^. Samples were spread onto potato dextrose agar (PDA, Difco, #213400) containing chloramphenicol (25 µg/mL) and incubated at 25 °C for 7 days. Subsequent culturing was also carried out on PDA medium (unless noted otherwise).

### Morphological analysis

To characterize colony morphology, the fungal strain FJI-L2-BK-P2 was streaked onto PDA and oatmeal agar (OMA, Difco, #255210) and incubated at 25 °C. Colony diameter (in mm), structure, pigmentation, and other morphological characteristics were recorded after 14 days and 21 days. Photographs of the colonies were taken using a smartphone camera (Samsung Galaxy A32, Model No. SM-A325M/DS). The slide culture technique (~ 30 days growth on PDA) was used to observe cell morphology by employing bright-field light microscopy with DIC^54^. The DIC images were generated on a Nikon Ti2-E inverted microscope with Nikon NIS Elements software. In addition, to gain more morphological insight phase contrast microscopy was used on a loopful of scraped mycelium mixed with a drop of water or lactophenol cotton blue (Z68, Hardy Diagnostics). Phase contrast images were generated on an Olympus BX53 microscope equipped with an Olympus DP25 camera and Olympus cellSens software. Measurements of fungal morphological structures were taken with either the Olympus cellSens or Nikon NIS Elements software.

### Scanning electron microscopy

Sample preparation and scanning electron microscopy (SEM) were performed as described previously^28^. In brief, fungal samples were obtained from cultures cultivated on PDA plates. These samples were then immersed in chilled 2.5% glutaraldehyde (Ted Pella Inc.) solution in 0.1 M sodium cacodylate buffer (Sigma–Aldrich) and incubated at 4 °C for 1 h. Subsequently, the samples were subjected to three washes with 0.1 M sodium cacodylate buffer to remove any excess fixative. Dehydration of the samples was carried out using isopropyl alcohol (IPA) with incremental concentrations from 50 to 100% (50%, 70%, 80%, 90%, 95%, and three times 100%). Following dehydration, the samples were stored at 4 °C in 100% IPA. As previously described^55^, critical point drying was performed to prepare the samples, which were then subjected to SEM using an FEI Quanta 200F scanning electron microscope (Thermo Fisher Scientific).

### Internal transcribed spacer (ITS)-based fungal identification

Initial identification of the fungus was performed by sequencing the ITS amplicon. Fungal biomass grown on PDA plates was collected and genomic DNA (gDNA) extracted with a Maxwell-16 MDx automated system, following the manufacturer’s instructions (Promega). The ITS region was amplified by polymerase chain reaction (PCR) with primers ITS 1F (5ʹ-CTT GGT CAT TTA GAG GAA GTA A-3′)^56^, and Tw13 (5′-GGT CCG TGT TTC AAG ACG-3′)^57^. The PCR conditions and sample preparation steps for sequencing were described elsewhere^58^. The ITS sequences were also included in the MLSA-based analysis.

### Whole genome sequencing

To sequence the genome of FJI-L2-BK-P2, gDNA was extracted from 1 g of wet biomass scraped from a PDA plate, using the ZymoBIOMICS MagBead DNA kit (Zymo Research) and a Precellys homogenizer (Bertin Technologies). Quality of genomic DNA was verified by gel electrophoresis and quantified by spectrophotometric measurement (Nanophotometer NP80 Mobile, Implen). Library preparation followed the Illumina Nextera Flex Protocol (Illumina document # 1000000025416 v07). The DNA was used for library preparation, and 5 to 12 cycles of PCR amplification were carried out to normalize the output depending on the input DNA concentration. The amplified genomic DNA fragments were indexed and pooled in a 384-plex configuration with dual-index adapters. WGS was performed on a NovaSeq 6000 S4 flow cell PE 2 × 150 platform with a paired-end module.

The genome was assembled after filtering with NGS QC Toolkit v2.3 for high-quality (HQ) vector and adaptor-free reads (cutoff read length for HQ 80%; cutoff quality score 20)^59^. The filtered reads were used with the Automatic Assembly for the Fungi (AAFTF v 0.3.3) pipeline^60^, as follows: (i) raw Illumina reads were trimmed using BBDuk module of BBMap (v 38.95*)*^61^; (ii) contaminant reads were removed using BBMap; (iii) the polished reads were assembled, using SPAdes v 3.15.4; (iv) contaminant contigs were identified and purged with vectrim and sourpurge steps, using the nucleotide Basic Local Alignment Search Tool (BLASTN)^62^; (v) duplicated contigs were identified and removed, using minimap2^63^; (vi) assembled contigs were polished with the Pilon v1.24 software^64^; (vii) the final scaffolds were sorted by length and the fasta headers renamed for deposit at the National Center for Biotechnology Information (NCBI). The assembly quality was verified with QUAST 5.1.0^65^.

For *Knufia obscura* CBS 148926^T^, gDNA was extracted from PDA-grown mycelium, using the DNeasy PowerSoil Kit (Qiagen) and following manufacturer’s protocol. Oxford Nanopore Technologies sequencing was performed on a GridION MK1 sequencer using a R10.4flow cell (FLO-MIN114) and a library synthesized from Q20 + EA (early access) ligation reagents (SQK-LSK114). The raw reads were base-called using MinKNOW v29.10.8 with a mean quality score of 16.3 and a mode of 18.03. All reads went through base calling with Guppy v5.1.13^66^. The genome was assembled with Flye assembler (version 2.9.2) by setting up the parameters g-31.5m and i-2. Assembly quality and genome features were verified with QUAST 5.1. 0^65^.

### Multi locus sequence analysis (MLSA)

We used the MLSA approach for the phylogenetic characterization of the two fungal strains. Initially, ITS amplicon sequences were used to determine preliminary genus assignment using NCBI BLAST^62^. After WGS was available, we extracted the desired nuclear markers according to the *Chaetothyriales* MLSA scheme from each genome, using a python wrapper script with the BLASTN tool^62^. Once the target genes were located and extracted from each genome, we performed a BLASTN analysis against the NCBI nucleotide database to confirm the homology with other *Chaetothyriales* species^62^. The gene sequences of ITS, LSU, translational elongation factor (*tef*1)*,* RNA polymerase I subunit (*rpb*1) and ß-tubulin were retrieved from various *Trichomeriaceae* species (Table S4), and *Arthrocladium fulminans* CBS 136243 was used as outgroup to generate the phylogenetic tree^67^. Individual alignments for each genetic marker were generated by Practical Alignments with the Sate and TrAnsitivity (PASTA) software^68^. Next, we used ClipKIT to trim uninformative sites from the DNA alignments according to the smart-gap mode^69^. Species tree inference was reconciliated from these alignments using the concatenation approach via IQ-TREE v 2.1.1^70^. The best DNA substitution model was inferred with the ModelFinder approach^71^, and branch support was calculated using ultrafast bootstraps and an SH-aLRT^72,73^. The tree topologies were then visualized with FigTree v 1.4.4^74^.

### Genome annotation

The genomes of the two *K. obscura* strains were annotated with the funannotate v1.8.1 pipeline^75^. We masked the repetitive DNA sequences using TANTAN via the funannotate mask command^76^. We predicted gene structure and content using different evidence-based and ab initio gene prediction algorithms with the funannotate predict command. Briefly, the steps funannotate performs start with using BUSCO (*Chaetothyriales*_odb10 database) to find conserved gene models for training the ab initio predictors Augustus^77^, glimmerhmm^78^, and SNAP^79^. We also used GeneMark-ES gene finder in iterative unsupervised mode, using the option for fungal genomes. Next, funannotate computed consensus gene models with EVidenceModeler to obtain a weighted consensus gene structure^79^; all predictors had weight = 1, but Augustus HiQ models were set to weight = 2. Gene models with less than 50 amino acids (aa) in length or identified as transposable elements were removed. Funannotate used tRNAscan-SE to predict tRNAs^80^. The two *K. obscura* strains’ annotation also applied previous functional analysis from Interproscan^81^, Eggnog^82^, Pfam^83^, CAZYme^84^, MEROPS^85^, BUSCO^77^, and Secreted proteins^86^. Telomers were identified based on repeat monomer pattern ([TAA[C]+]) occurring in the end of scaffolds with minimum number of 2. Gene Ontology (GO) terms were appended to the final annotation file in ASN.1 format for deposit into NCBI GenBank. The assembled genomes were submitted to fungiSMASH, the fungal-specific pipeline for antiSMASH^87^, for prediction of the presence of biosynthetic gene clusters. The predicted proteomes of the two *Knufia obscura* strains were compared using the OrthoVenn3 tool^88^. The shared and unique ortholog protein clusters were submitted to KEGG analysis revealing common and unique biological features of those two close related taxa^89–91^. Gene gain and loss were also evaluated using the CAFE5 pipeline^92^. GO enrichment analysis were calculated using the log2 Ratio of Classes option as a metric and selecting the significantly enriched terms.

### Extraction of gene information from GenBank (.gbk) files

A comprehensive methodology to extract and analyze gene information from GenBank files was developed by using Python (version 3.x), Biopython (version 1.78)^93^, and Pandas (version 1.2.x) for automated data processing^94^. GenBank files were parsed using the SeqIO module from Biopython to extract gene and coding sequence (CDS) features, including gene names, product descriptions, and genomic locations. We structured this information into tabular datasets, focusing on removing rows lacking gene names and entries corresponding to hypothetical proteins to ensure the analysis focused on genes with known or predicted functions. Further data cleanup steps were implemented to refine the datasets, including renaming columns with unique identifiers, merging datasets based on gene names using an outer join to preserve all unique gene entries, and creating a consolidated 'Gene Name' column. Additional filters were used to eliminate rows with NaN values in specific columns, remove duplicates in specific gene identifiers, and exclude entries without product/proteins names. The final, cleaned dataset was exported to an Excel spreadsheet with Pandas' to_excel method^94^.

### Whole genome-based phylogenetic tree

To gain a comprehensive understanding of the phylogenetic positioning of the two strains of *K. obscura*, we conducted a phylogenomic analysis utilizing conserved genes within the fungal kingdom. The PHYling pipeline was employed for this purpose^95^. Protein models representing the *Trichomeriaceae* family were obtained from the Mycocosm portal^96^. For genomes lacking annotations, contigs from each species were retrieved (Table S5), and gene models were predicted using the same approach as described earlier for both *K. obscura* strains. Subsequently, the hmmsearch tool, available in the HMMER v3.3.2 software, was employed to identify homologous sequences present in the fungi_odb10 database. These sequences serve as a universal benchmark for evolutionary investigations in fungi. Individual protein alignments were constructed using the hmmbuild function of HMMER v3.3.2, and any erroneous positions in the alignments were removed using the ClipKIT v1.3.0 tool with the smartgap function. A phylogenetic tree was then generated using the IQTREE2 software^70^. In summary, a species tree was created based on 686 protein markers. Additionally, individual gene trees were generated to calculate concordance factors for each branch. We also determined ultrafast bootstraps and conducted an SH-aLRT to assess branch support^72^. Finally, the resulting tree was visualized using FigTree v1.4.4.

### UV-C resistance evaluation

The radiation resistance in strain FJI-L2-BK-P2 was evaluated using methods previously employed for the strains *Pasadenomyces melaninifex* and *Floridaphiala radiotolerans* except for the different cfu counts per coupon and wide range of UV-C doses^28^. Round aluminum coupons (Diameter = 13 mm, thickness 2 mm) loaded with a suspension (150 μL) of arthroconidia and chlamydospores (153/150 μL of sterile water) were exposed to UV-C doses ranging from 1000 to 9999 J/m^2^ (1000, 2000, 3000, 5000, 7000, 8000, and 9999 J/m^2^) using a UV Crosslinker CL-1000 (UVP Inc). For each radiation dose point, the experiment was conducted with three technical replicates. After radiation exposures, the test coupon was placed into potato dextrose broth and incubated at 25 °C for 7 days. The survival of fungi was recorded as a binary outcome (yes/no).

### Informed consent statement

This manuscript was prepared as an account of work sponsored by NASA, an agency of the US Government. The US Government, NASA, California Institute of Technology, Jet Propulsion Laboratory, and their employees make no warranty, expressed or implied, or assume any liability or responsibility for the accuracy, completeness, or usefulness of information, apparatus, product, or process disclosed in this manuscript, or represents that its use would not infringe upon privately held rights. The use of, and references to any commercial product, process, or service does not necessarily constitute or imply endorsement, recommendation, or favoring by the US Government, NASA, California Institute of Technology, or Jet Propulsion Laboratory. Views and opinions presented herein by the authors of this manuscript do not necessarily reflect those of the US Government, NASA, California Institute of Technology, or Jet Propulsion Laboratory, and shall not be used for advertisements or product endorsements.

### Supplementary Information

Supplementary Table S1.

Supplementary Table S2.

Supplementary Table S3.

Supplementary Table S4.

Supplementary Table S5.

## Data Availability

This Whole Genome Shotgun project for strain CBS 148926^T^ and FJI-L2-BK-P2 has been deposited at DDBJ/ENA/GenBank under the accessions JAVHJV000000000 and JAKLMC000000000. The versions described in this paper are version JAVHJV010000000 and version JAKLMC010000000.
